# Synthesis of Schiff and Mannich Bases of Isatin Derivatives with 4-Amino-4,5-Dihydro-1H-1,2,4-Triazole-5-Ones

**DOI:** 10.3390/molecules13092126

**Published:** 2008-09-10

**Authors:** Olcay Bekircan, Hakan Bektas

**Affiliations:** 1 Department of Chemistry, Karadeniz Technical University, 61080 Trabzon, Turkey; 2 Department of Chemistry, Giresun University, 28049 Giresun, Turkey; E-mail: hakanbektas@ktu.edu.tr

**Keywords:** Ethyl imidate hydrochloride, ethoxycarbonyl hydrazones, 4-amino-1,2,4-triazole-5-one, isatin, Schiff bases, Mannich bases.

## Abstract

Ethyl imidate hydrochlorides **1** were prepared by passing HCl gas through solutions of substituted benzyl cyanides and absolute ethanol. Ethoxycarbonylhydrazones **2** were synthesized from the reaction of compounds **1** with ethyl carbazate. Treatment of **2** with hydrazine hydrate leads to the formation of substituted 4-amino-4,5-dihydro-1*H*-1,2,4-triazole-5-ones **3**. Isatin and 5-chloroisatin were added to **3** to form Schiff bases **4** and *N*-Mannich bases **5** of these compounds were synthesized by reacting with formaldehyde and piperidine. Their chemical structures were confirmed by means of IR, ^1^H- and ^13^C-NMR data and by elemental analysis.

## Introduction

Schiff bases are used as substrates in the preparation of a number of industrial and biologically active compounds via ring closure, cycloaddition and replacement reactions [1]. Moreover, Schiff bases derived from various heterocycles have been reported to possess cytotoxic [2], anticonvulsant [3], antiproliferative [4], antimicrobial [5], anticancer [6], and antifungal activities [7]. Mannich bases have gained importance due to their application in pharmaceutical chemistry. They have been encountered with antibacterial [8], anticancer [9], analgesic and anti-inflammatory [10], anticonvulsant [11], antimalarial [12], antiviral [13], and CNS depressant activities [14]. Isatin, chemically known as 1*H*-indole-2,3-dione, has become a popular topic due to its various uses. The chemistry of isatin and its derivatives is particularly interesting because of their potential application in medicinal chemistry. Isatins are very important compounds due to their antifungal properties [15]. Schiff and Mannich bases of isatin derivatives are reported to show variety of biological activities like antibacterial [16], antifungal [17], anticonvulsant [18], anti HIV [19], antidepressant [20], and antiinflammatory [21] activities. Similarly, 1,2,4-triazoles and their derivatives play important roles in medicinal, agricultural and industrial fields [22,23,24,25]. *N*-bridged heterocyclic derivatives derived from 1,2,4-triazoles show varied biological activities such as antimicrobial [26], anticonvulsant [27], anticancer [28], analgesic [29], anti HIV [30], and anti-inflammatory properties [31].

These biological data prompted us to synthesize new isatin derivatives bearing 1,2,4-triazole ring and the newly synthesized compounds were characterized by elemental analysis, IR, ^1^H- and ^13^C-NMR spectral data.

## Results and Discussion

**Scheme 1:**
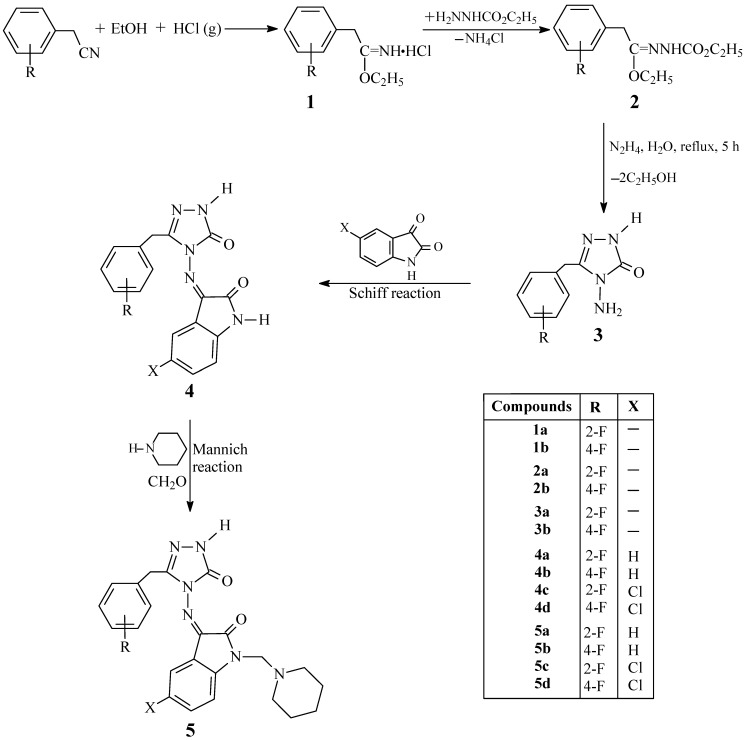
Synthetic pathway for preparation of compounds **1** and **5**.

In the present study, ethyl imidate hydrochlorides **1** were prepared by passing HCl gas through solutions of *o*- or *p*-fluorobenzyl cyanide in absolute ethanol, followed by precipitation with ether. Ester ethoxycarbonyl hydrazones **2** were synthesized by the reactions of alkyl imidate hydrochlorides **1** with ethyl carbazate. Treatment of compounds **2** with hydrazine hydrate in water resulted in the formation of 3-*o*-fluorobenzyl-4-amino-4,5-dihydro-1*H*-1,2,4-triazole-5-one (**3a**) and 3-*p*-fluorobenzyl-4-amino-4,5-dihydro-1*H*-1,2,4-triazole-5-one (**3b**), respectively. The substituted 4-amino-4,5-dihydro-1H-1,2,4-triazoles **3** were condensed with isatin derivatives in the presence of a few drops of glacial acetic acid as a catalyst to produce Schiff bases **4** in rather good yields. Mannich base formation can take place at both the isatin and triazole NH protons, but it is known that the isatin NH proton is more active than the triazole one [32]. In addition, after the Mannich reactions, the ^1^H- NMR spectra of compounds **5** show NH proton signals at around δ 11.00 ppm, which correspond to a triazole NH, so we conclude that in the current study, Mannich bases were formed by condensing the acidic imino group of isatin with formaldehyde and piperidine, instead of the triazole NH proton (Scheme 1). 

Compounds **2** showed absorption bands at around 3277, 1720, and 1659 cm^-1^ regions, resulting from the NH, C=O, and C=N functions, respectively. Compounds **3** showed two peaks at around 3220 and 3165 cm^-1^ due to asymmetric and symmetric vibrations of the primary amino group. Compounds **4** and **5** showed two separate bands belonging to triazole indole C=O functions in the 1733-1750 and 1698-1710 cm^-1^ regions, and C=N stretching vibrations at around 1650 cm^-1^. In the IR spectra of **5**, triazole NH stretching vibrations were around 3200 cm^-1^. The characteristic NH protons of compounds **2** were detected around δ 9.00 ppm. The characteristic NH_2_ protons of compounds **3** were detected around δ 5.00 ppm. The ^1^H-NMR spectra of **4** displayed the NH protons of the triazole moiety (δ 11.07-11.17 ppm) and the indole NH proton (δ 12.09-12.53) as two separate singlets. Compounds **5** displayed characteristic >N-CH_2_-N< signals at around δ 4.36 ppm (s, 2H, CH_2_).

In the ^13^C-NMR spectra of compounds **2a-b** characteristic C=O signals appeared at around δ 171.00 ppm. The triazole C-3 and triazole C-5 signals of compounds **3a-b** were recorded at δ 149.38 ppm (C-3) and δ 156.33 ppm (C-5). Characteristic triazole and indole C=O and the C=N carbon signals of compounds **4a-d** were recorded at around δ 150 ppm, δ 165 ppm, and δ 150 ppm, respectively. In the ^13^C-NMR spectra of compounds **5a-d** characteristic >N-CH_2_-N< signals belonging to the Mannich bases were observed at around δ 171.00 ppm. 

## Conclusions

It is known that the heterocycle compounds containing both 1,2,4-triazole and isatin rings have diverse pharmacologic properties [15,16,17,18,19,20,25,26,27,28,29,30,31,33]. A general and convenient method was established for the synthesis of new heterocyclic compounds bearing these two rings. Thus, two new ester ethoxycarbonyl hydrazones **2**, two new amino compounds **3**, and eight new Schiff and Mannich bases of isatin and 5-chloro isatin with substituted 4-amino-4,5-dihydro-1H-1,2,4-triazole-5-ones **4-5** were synthesized in good yields. Compounds **4-5** are expected to exhibit biological activities.

## Experimental

### General

Melting points were determined on a Barnstead Electrothermal melting point apparatus and are uncorrected. ^1^H-NMR and ^13^C-NMR spectra (δ, ppm) were recorded in DMSO-d_6_ solutions on a Varian-Mercury 200 MHz spectrometer using tetramethylsilane as the internal reference. The IR spectra (υ, cm^-1^) were obtained with a Perkin-Elmer 1600 FTIR spectrometer in KBr pellets. Elemental analyses were performed on a ECS 4010 Elemental Combustion System. The starting compounds, alkyl imidate hydrochlorides **1a-b**, were synthesized by previously reported routes [34,35]. The necessary chemicals were purchased from Merck and Fluka companies.

### Synthesis of Ethoxycarbonyl Hydrazones **2a-b**

The corresponding ethyl imidate hydrochlorides (**1a-b**, 0.01 mol) was dissolved in absolute ethanol (50 mL) with ice-bath cooling, and ethyl carbazate (0.01 mol) dissolved in absolute ethanol (50 mL) was then added to this solution. After stirring for 6 h at 0-5 °C, the precipitate was filtered to remove the ammonium chloride which separated from the solution and the filtrate was evaporated at 30-35 °C under reduced pressure. The solid residue, after drying in a dessicator, was recrystallized from petroleum ether to yield compounds **2a-b**.

*Ethyl o-fluorophenylacetate ethoxycarbonyl hydrazone* (**2a**): Yield 82%, m.p. 48-49 °C; IR υ (cm^-1^): 3277 (NH), 1720 (C=O), 1650 (C=N), 758 (1,2-disubstituted phenyl); ^1^H-NMR δ (ppm): 1.10-1.20 (m, 6H, 2CH_3_), 3.74 (s, 2H, benzyl CH_2_), 3.96-4.09 (m, 4H, 2CH_2_), 7.11-7.29 (m, 4H, Ar-H), 9.37 (s, 1H, NH); ^13^C-NMR δ (ppm): 15.70 (O=COCH_2_CH_3_), 16.38 (OCH_2_CH_3_), 29.55 (benzyl CH_2_), 61.96 (O=COCH_2_CH_3_), 63.59 (OCH_2_CH_3_), Ar-C: [115.58, 124.35, 125.00, 126.32, 130.79, 156.19], 163.11 (C=N), 171.92 (C=O); Calculated (%) for C_13_H_17_FN_2_O_3_ (268.29); C: 58.20, H: 6.39, N: 10.44, found (%); C: 58.18, H: 6.40, N: 10.45.

*Ethyl p-fluorophenylacetate ethoxycarbonyl hydrazone* (**2b**): Yield 80%, m.p. 58-59 °C; IR υ (cm^-1^): 3226 (NH), 1724 (C=O), 1659 (C=N), 837 (1,4-disubstituted phenyl); ^1^H-NMR δ (ppm): 1.11-1.18 (m, 6H, 2CH_3_), 3.45 (s, 2H, benzyl CH_2_), 4.00-4.06 (m, 4H, 2CH_2_), 7.07 (d, 2H, J= 8.80 Hz, Ar-H), 7.21 (d, 2H, J= 8.80 Hz, Ar-H), 9.08 (s, 1H, NH); ^13^C-NMR δ (ppm): 14.72 (O=COCH_2_CH_3_), 15.22 (OCH_2_CH_3_), 21.39 (benzyl CH_2_), 60.44 (O=COCH_2_CH_3_), 60.93 (OCH_2_CH_3_), Ar-C: [115.55 (2C), 130.85 (2C), 133.19, 160.39], 162.79 (C=N), 171.87 (C=O); Calculated (%) for C_13_H_17_FN_2_O_3_ (268.29); C: 58.20, H: 6.39, N: 10.44, found (%);C: 58.22, H: 6.40, N: 10.47.

### Synthesis of Amino Compounds **3a-b**

Compounds **2** (0.01 mol) were added to a solution of hydrazine hydrate (0.01 mol) in water (50 mL) and the mixture was refluxed for 5 h. On cooling, a precipitate was formed. This product was filtered and, after drying, was recrystallized from an appropriate solvent to give compounds **3a-b**.

*3-o-Fluorobenzyl-4-Amino-4,5-dihydro-1H-1,2,4-triazole-5-one* (**3a**): Recrystallized from water; Yield 87%; m.p. 158-159 °C; IR υ (cm^-1^): 3316 (NH), 3220-3164 (NH_2_), 1726 (C=O), 1652 (C=N), 764 (1,2-disubstituted phenyl); ^1^H-NMR δ= 3.91 (s, 2H, benzyl CH_2_), 5.21 (s, 2H, NH_2_), 7.11-7.36 (m, 4H, Ar-H), 11.44 (s, 1H, NH); ^13^C-NMR δ= 25.87 (benzyl CH_2_), Ar-C: [116.68, 124.65, 121.11, 130.50, 132.92, 164.60], 149.38 (triazole C-3), 156.35 (triazole C-5); Calculated (%) for C_9_H_9_FN_4_O (208.20); C: 51.92, H: 4.36, N: 26.91, found (%);C: 51.82, H: 4.36, N: 26.96.

*3-p-Fluorobenzyl-4-Amino-4,5-dihydro-1H-1,2,4-triazole-5-one* (**3b**): Recrystallized from ethanol-water (1:2); Yield 82%; m.p. 182-183 °C; IR υ (cm^-1^): 3318 (NH), 3223-3165 (NH_2_), 1729 (C=O), 1644 (C=N), 844 (1,4-disubstituted phenyl); ^1^H-NMR δ= 3.86 (s, 2H, benzyl CH_2_), 4.60 (s, 2H, NH_2_), 7.13 (d, 2H, J= 6.4 Hz, Ar-H), 7.32 (d, 2H, J= 6.4 Hz, Ar-H), 11.43 (s, 1H, NH); ^13^C-NMR δ= 31.61 (benzyl CH_2_), Ar-C: [116.60 (2C), 132.31 (2C), 134.01, 165.27], 149.38 (triazole C-3), 156.33 (triazole C-5); Calculated (%) for C_9_H_9_FN_4_O (208.20); C: 51.92, H: 4.36, N: 26.91, found (%);C: 51.90, H: 4.35, N:26.94.

### Synthesis of Schiff Bases **4a-d**

Equimolar quantities (0.01 mol) of isatin or 5-chloroisatin and the corresponding amino compound **3a-b** were dissolved in warm ethanol (50 mL) containing glacial acetic acid (0.5 mL). The reaction mixture was refluxed for 4 h and then kept at room temperature overnight. The resultant solid was washed with dilute ethanol, dried and recrystallized from ethanol-water (1:2) mixture to afford compounds **4a-d**.

*3-[3-(o-Fluorobenzyl)-5-oxo-4,5-dihydro-1,2,4-triazol-1-yl]-iminoisatin* (**4a**): Yield 78%; obtained as yellowish crystals; m.p. 278-279 °C; IR υ (cm^-1^): 3182, 3087 (NH), 1750 (triazole C=O), 1709 (isatin C=O), 754 (1,2-disubstituted phenyl); ^1^H-NMR δ= 4.02 (s, 2H, benzyl CH_2_), Ar-H: [ 6.88-6.99 (m, 1H), 7.02-7.15 (m, 4H), 7.20-7.36 (m, 2H), 7.43-7.50 (m, 1H) ], 11.07 (s, 1H, triazole NH), 12.11 (s, 1H, isatin NH) ppm; ^13^C-NMR δ= 27.03 (benzyl CH_2_), Ar-C: [116.72, 123.4, 123.73, 126.07, 130.98, 159.75], indole-C: [ 112.92, 117.56, 124.12, 131.04, 133.21, 137.27 (C=N), 146.66, 165.00 (C=O)], 147.96 (triazole C-3), 150.94 (triazole C-5) ppm; Calculated (%) for C_17_H_12_FN_5_O_2_ (337.31); C: 60.53, H: 3.59, N: 20.76, found (%);C: 60.54, H: 3.81, N: 20.64.

*3-[3-(p-Fluorobenzyl)-5-oxo-4,5-dihydro-1,2,4-triazol-1-yl]-iminoisatin* (**4b**): Yield 82%; obtained as yellow-orange crystals; m.p. 253-254 °C; IR υ (cm^-1^): 3175, 3089 (NH), 1733 (triazole C=O), 1710 (isatin C=O), 842 (1,4-disubstituted phenyl); ^1^H-NMR δ= 4.00 (s, 2H, benzyl CH_2_), Ar-H: [ 6.90-7.09 (m, 5H), 7.25-7.32 (m, 2H), 7.42-7.50 (m, 1H) ], 11.15 (s, 1H, triazole NH), 12.20 (s, 1H, isatin NH) ppm; ^13^C-NMR δ= 32.51 (benzyl CH_2_), Ar-C: [116.61 (2C), 132.52 (2C), 132.99, 159.27], indole-C: [112.91, 117.03, 124.11, 129.71, 132.46, 137.26 (C=N), 147.65, 165.01 (C=O)], 147.91(triazole C-3), 150.73 (triazole C-5) ppm; Calculated (%) for C_17_H_12_FN_5_O_2_ (337.31); C: 60.53, H: 3.59, N: 20.76, found (%);C: 60.54, H: 3.58, N: 20.74.

*3-[3-(o-Fluorobenzyl)-5-oxo-4,5-dihydro-1,2,4-triazol-1-yl]-imino-5-chloro-isatin* (**4c**): Yield 85%; obtained as orange crystals; m.p. 302-303 °C; IR υ (cm^-1^): 3203, 3080 (NH), 1747 (triazole C=O), 1701 (isatin C=O), 755 (1,2-disubstituted phenyl); ^1^H-NMR δ= 4.04 (s, 2H, benzyl CH_2_), Ar-H: [6.93-6.96 (m, 1H), 7.10-7.27 (m, 2H), 7.29-7.37 (m, 2H), 7.54-7.64 (m, 2H)], 11.17 (s, 1H, triazole NH), 12.09 (s, 1H, isatin NH) ppm; ^13^C-NMR δ= 26.95 (benzyl CH_2_), Ar-C: [116.73, 123.47, 124.29, 126.07, 130.99, 159.99], indole-C: [114.56, 122.30, 128.38, 131.07, 133.11, 136.36 (C=N), 145.46, 162.87 (C=O)], 145.94 (triazole C-3), 150.46 (triazole C-5) ppm; Calculated (%) for C_17_H_11_ClFN_5_O_2_ (371.76); C: 54.92, H: 2.98, N: 18.84, found (%);C: 54.94, H: 2.98, N:18.80.

*3-[3-(p-Fluorobenzyl)-5-oxo-4,5-dihydro-1,2,4-triazol-1-yl]-imino-5-chloro-isatin (**4d**)*: Yield 80%; obtained as orange crystals; m.p. 194-195 °C; IR υ (cm^-1^): 3193, 3078 (NH), 1750 (triazole C=O), 1698 (isatin C=O), 841 (1,4-disubstituted phenyl); ^1^H-NMR δ= 3.96 (s, 2H, benzyl CH_2_), Ar-H: [6.93-7.13 (m, 3H), 7.28-7.35 (m, 2H), 7.56-7.66 (m, 2H)], 11.15 (s, 1H, triazole NH), 12.53 (s, 1H, isatin NH) ppm; ^13^C-NMR δ= 31.61 (benzyl CH_2_), Ar-C: [115.61 (2C), 132.48 (2C), 133.87, 155.32], indole-C: [117.03, 120.92, 125.92, 128.04, 132.33, 134.09 (C=N), 139.10, 160.92 (C=O)], 149.38 (triazole C-3), 150.93 (triazole C-5) ppm; Calculated (%) for C_17_H_11_ClFN_5_O_2_ (371.76); C: 54.92, H: 2.98, N: 18.84, found (%);C: 54.96, H: 2.66, N:18.72.

### Synthesis of Mannich Bases **5a-d**

The corresponding Schiff bases **4a-d** (0.002 mol) were dissolved in absolute ethanol (100 mL). Then formaldehyde (37%, 0.5 mL) and piperidine (0.002 mol) were added dropwise with vigorous stirring. After combining all reagents, the reaction mixture was stirred at room temperature for 12 h. The mixture was cooled, the solid product was filtered and washed with petroleum ether. The solid that separated was recrystallized from ethanol-dioxane (1:2) to yield the title compounds **5a-d**.

*1-Piperidinomethyl-3-[3-(o-fluorobenzyl)-5-oxo-4,5-dihydro-1,2,4-triazol-1-yl]-iminoisatin* (**5a**): Yield 72%; obtained as yellow crystals; m.p. 115-116 °C; IR υ (cm^-1^): 3247 (NH), 2936 (aliphatic CH_2_), 1745 (triazole C=O), 1709 (isatin C=O), 757 (1,2-disubstituted phenyl); ^1^H-NMR δ= 1.45 (br, s, 6H, piperidine 3CH_2_), 2.51 (br, s, 4H, piperidine 2CH_2_), 4.01 (s, 2H, benzyl CH_2_), 4.36 (s, 2H, N-CH_2_-N), 6.91-7.11 (m, 3H, Ar-H), 7.26-7.30 (m, 2H, Ar-H), 7.50-7.70 (m, 3H, Ar-H), 11.16 (s, 1H, triazole NH) ppm; ^13^C-NMR δ= 25.40 (piperidine C), 27.12 (piperidine 2C), 31.28 (benzyl CH_2_), 52.93 (piperidine 2C), 69.32 (N-CH_2_-N), Ar-C: [115.65, 125.40, 125.99, 127.74, 128.63, 161.00], indole-C: [117.20, 120.98, 126.04, 129.41, 130.99, 139.07 (C=N), 146.88, 164.54 (C=O)], 147.27 (triazole C-3), 151.04 (triazole C-5) ppm; Calculated (%) for C_23_H_23_FN_6_O_2_ (434.47); C: 63.58, H: 5.34, N: 19.34, found (%);C: 63.19, H: 5.33, N:19.54.

*1-Piperidinomethyl-3-[3-(p-fluorobenzyl)-5-oxo-4,5-dihydro-1,2,4-triazol-1-yl]-iminoisatin* (**5b**): Yield 65%; obtained as yellow crystals; m.p. 141-142 °C; IR υ (cm^-1^): 3236 (NH), 2936 (aliphatic CH_2_), 1740 (triazole C=O), 1705 (isatin C=O), 856 (1,4-disubstituted phenyl); ^1^H-NMR δ= 1.46 (br, s, 6H, piperidine 3CH_2_), 2.50 (br, s, 4H, piperidine 2CH_2_), 4.00 (s, 2H, benzyl CH_2_), 4.59 (s, 2H, N-CH_2_-N), 6.93-7.07 (m, 5H, Ar-H), 7.23-7.30 (m, 2H, Ar-H), 7.42-7.50 (m, 1H, Ar-H), 11.11 (s, 1H, triazole NH) ppm; ^13^C-NMR δ= 25.39 (piperidine C), 27.33 (piperidine 2C), 32.40 (benzyl CH_2_), 52.75 (piperidine 2C), 69.37 (N-CH_2_-N), Ar-C: [116.62 (2C), 129.99 (2C), 132.36, 159.77], indole-C: [117.09, 123.91, 124.45, 129.37, 132.53, 137.52 (C=N), 148.09, 164.46 (C=O)], 149.55 (triazole C-3), 154.90 (triazole C-5) ppm; Calculated (%) for C_23_H_23_FN_6_O_2_ (434.47); C: 63.58, H: 5.34, N: 19.34, found (%);C: 63.58, H: 5.36, N:19.36.

*1-Piperidinomethyl-3-[3-(o-fluorobenzyl)-5-oxo-4,5-dihydro-1,2,4-triazol-1-yl]-imino-5-chloroisatin* (**5c**): Yield 64%; obtained as reddish-orange crystals; m.p. 155-156 °C; IR υ (cm^-1^): 3224 (NH), 2935 (aliphatic CH_2_), 1748 (triazole C=O), 1708 (isatin C=O), 756 (1,2-disubstituted phenyl); ^1^H-NMR δ= 1.22 (br, s, 6H, piperidine 3CH_2_), 2.26 (br, s, 4H, piperidine 2CH_2_), 3.82 (s, 2H, benzyl CH_2_), 4.35 (s, 2H, N-CH_2_-N), 6.99-7.04 (m, 5H, Ar-H), 7.28-7.34 (m, 2H, Ar-H), 11.13 (s, 1H, triazole NH) ppm; ^13^C-NMR δ= 25.30 (piperidine C), 27.40 (piperidine 2C), 31.24 (benzyl CH_2_), 52.50 (piperidine 2C), 69.67 (N-CH_2_-N), Ar-C: [116.57, 124.06, 124.16, 125.98, 130.64, 159.66], indole-C: [117.09, 123.75, 130.49, 132.94, 133.00, 139.73 (C=N), 146.26, 164.55 (C=O) ], 146.33 (triazole C-3), 154.54 (triazole C-5) ppm; Calculated (%) for C_23_H_22_ClFN_6_O_2_ (468.92); C: 58.91, H: 4.73, N: 17.92, found (%);C: 58.64, H: 4.70, N: 17.86.

*1-Piperidinomethyl-3-[3-(p-fluorobenzyl)-5-oxo-4,5-dihydro-1,2,4-triazol-1-yl]-imino-5-chloroisatin* (**5d**): Yield 67%; obtained as reddish-orange crystals; m.p. 126-127 °C; IR υ (cm^-1^): 3251 (NH), 2937 (aliphatic CH_2_), 1748 (triazole C=O), 1708 (isatin C=O), 846 (1,4-disubstituted phenyl); ^1^H-NMR δ= 1.46 (br, s, 6H, piperidine 3CH_2_), 2.51 (br, s, 4H, piperidine 2CH_2_), 4.02 (s, 2H, benzyl CH_2_), 4.36 (s, 2H, N-CH_2_-N), 6.91-7.11 (m, 3H, Ar-H), 7.26-7.30 (m, 2H, Ar-H), 7.50-7.70 (m, 2H, Ar-H), 11.16 (s, 1H, triazole NH) ppm; ^13^C-NMR δ= 25.40 (piperidine C), 27.26 (piperidine 2C), 32.11 (benzyl CH_2_), 52.69 (piperidine 2C), 69.45(N-CH_2_-N), Ar-C: [115.59 (2C), 125.97 (2C), 132.66, 160.99], indole-C: [117.10, 120.87, 125.40, 128.55, 132.45, 139.67 (C=N), 146.85, 164.87 (C=O)], 149.17 (triazole C-3), 151.07 (triazole C-5); Calculated (%) for C_23_H_22_ClFN_6_O_2_ (468.92); C: 58.91, H: 4.73, N: 17.92, found (%);C: 58.95, H: 4.71, N:17.95.
